# Exploring Dietary and Lifestyle Profiles in Colorectal Cancer Patients: Hypothesis-Generating Insights for Tertiary Prevention

**DOI:** 10.3390/cancers17162654

**Published:** 2025-08-14

**Authors:** Kamil Mąkosza, Janusz Wierzgoń, Małgorzata Muc-Wierzgoń, Sylwia Dzięgielewska-Gęsiak

**Affiliations:** 1 Doctoral School, Medical University of Silesia, 40-055 Katowice, Poland; 2 Department of Internal Diseases Propaedeutics and Emergency Medicine, Faculty of Public Health in Bytom, Medical University of Silesia, 41-902 Bytom, Poland; mwierzgon@sum.edu.pl (M.M.-W.); sgesiak@sum.edu.pl (S.D.-G.); 3 First Department of Oncological Surgery, National Institute of Oncology, 44-102 Gliwice, Poland; janusz.wierzgon@gliwice.nio.gov.pl

**Keywords:** exploring, diet, lifestyle, colorectal cancer patients, profiles, age, body mass index, education, tertiary prevention

## Abstract

**Background**: Colorectal cancer (CRC) is one of the most prevalent malignancies worldwide, particularly affecting the elderly. Tertiary prevention—focused on nutrition and lifestyle—may play an important role in improving treatment outcomes and quality of life. This exploratory study aimed to describe dietary and lifestyle behaviors among CRC patients, considering age, body mass index (BMI), and educational background as potential differentiating factors. **Methods**: A cross-sectional study was conducted among 202 CRC patients using a validated paper-based questionnaire. Sociodemographic data and health behaviors were analyzed using chi-square tests. **Results**: Age-related differences revealed that middle-aged patients consumed vegetables and legumes more frequently but also reported higher fast-food intake. Elderly individuals more often abstained from alcohol and tobacco. Overweight and obese participants were more likely to consume meat and sweets regularly and preferred frying over other cooking methods. Higher education was associated with lower rates of smoking and alcohol use. **Conclusions**: The findings suggest that dietary and lifestyle profiles among CRC patients may vary by age, BMI, and education. While causal relationships cannot be established, the results may help generate hypotheses for future research and support the development of individualized tertiary prevention strategies.

## Simple Summary

Colorectal cancer is one of the most common cancers, especially in older adults. Healthy eating habits and lifestyle choices may help patients during medical treatment and improve their daily well-being. This exploratory study examined how people diagnosed with colorectal cancer eat, whether they use tobacco or alcohol, and how they prepare their meals. It also looked at whether these behaviors appear to differ by age, body mass index (BMI), or education level. Younger patients tended to consume more vegetables but also more fast food, while older individuals more often abstained from alcohol and smoking. Patients with higher BMI reported more frequent consumption of fried foods and sweets. These findings may help generate new ideas for tailoring support strategies based on patients’ age, BMI, and education, with the goal of improving recovery and quality of life during cancer care.

## 1. Introduction

Colorectal cancer (CRC) remains one of the most frequently diagnosed malignancies worldwide and ranks among the leading causes of cancer-related morbidity and mortality in both men and women [1,2]. Despite considerable progress in screening and treatment, CRC continues to impose a significant public health burden—not only in terms of incidence and mortality, but also through its long-term impact on patients’ quality of life and functional capacity [3]. As noted by Parkin et al., “mortality is the product of the incidence and the fatality for a given cancer,” meaning that prevalence itself does not directly drive cancer-related mortality, but rather reflects the number of existing cases in a population [4].

Moreover, epidemiological studies have identified numerous non-modifiable and modifiable factors associated with CRC risk [5,6,7,8]. Non-modifiable factors include age, sex, race/ethnicity, inflammatory bowel diseases, genetic factors, and family history of colorectal cancer. Among these, age appears particularly important: more than 90% of colorectal cancer cases are diagnosed in people over the age of 50 [5,9,10], although recent data suggest a gradual increase in early-onset CRC in some regions [11].

While non-modifiable factors shape individual risk, lifestyle-related behaviors—many of which are modifiable—also play a central role in the prevention and management of CRC [12]. Large-scale studies consistently emphasize the importance of healthy dietary habits, regular physical activity, maintaining an appropriate BMI, adequate sleep, stress management, and avoiding harmful exposures such as tobacco, alcohol, and ultra-processed foods. Such behaviors have been associated not only with reduced CRC incidence, but also with better outcomes following diagnosis. Interventions targeting diet and physical activity in the post-diagnosis phase may support clinical recovery, reduce recurrence risk, and improve long-term well-being (secondary and tertiary prevention) [13,14,15,16,17].

Tertiary prevention refers to strategies aimed at mitigating the long-term effects of cancer and its treatment [18]. These strategies focus on preserving functional status, reducing recurrence risk, and minimizing complications associated with comorbid conditions. The overarching goal is not only to prolong survival but also to support quality of life and autonomy—particularly in older survivors, who often face age-related physiological changes and multiple chronic conditions. From a public health perspective, enhancing outcomes for CRC patients requires an integrated, person-centered approach that reflects the interaction of medical, functional, and sociodemographic determinants.

Among tertiary prevention strategies, nutrition plays a particularly important role [19,20,21,22,23]. Clinical guidelines from the European Society for Clinical Nutrition and Metabolism (ESPEN) recommend personalized nutritional planning based on gastrointestinal function, symptom burden, and comorbidities [24]. This may include adjustments in fiber intake, food texture, meal frequency, and energy/protein density. A protein intake of at least 1.0–1.5 g/kg/day is often recommended to preserve lean body mass, support immune competence, and improve treatment tolerance. Attention to fluid intake is also essential, especially in cases of dehydration or gastrointestinal losses [25,26]. Higher protein intakes—up to 2.0 g/kg/day—may be safe and beneficial for selected patients undergoing chemotherapy.

In specific clinical contexts—such as the postoperative period or among patients with ostomies—temporary dietary modifications (e.g., low-residue diets) may be necessary, with gradual reintroduction of fiber as tolerated [22,27]. During the survivorship phase, dietary recommendations from the World Cancer Research Fund (WCRF) and the American Cancer Society (ACS) promote a predominantly plant-based diet rich in vegetables, fruits, whole grains, and legumes, with limited consumption of red and processed meats, alcohol, and added sugars [27,28]. Although originally developed for cancer prevention, these guidelines are increasingly being adapted to post-treatment care, with modifications based on digestive function and nutritional risk.

Physical activity represents another important component of tertiary prevention. International recommendations from the American College of Sports Medicine (ACSM) and ACS advocate for minimizing sedentary behavior and engaging in light activity even during treatment, when feasible [29,30,31]. Following treatment, individualized programs combining aerobic and resistance training are encouraged to support physical functioning and overall health. Personalization, safety, and consistency are key principles in such interventions.

Despite growing interest in nutritional and lifestyle interventions in tertiary care, relatively little is known about how such behaviors may vary among CRC patients based on demographic or clinical characteristics [23,24,25,26,27]. To date, few studies have examined in depth stratified behavioral patterns, which may limit efforts to develop truly tailored approaches in survivorship care [28,29,32,33].

Given the increasing emphasis on personalized survivorship care in colorectal cancer, this study focused on three key stratification variables: age, body mass index (BMI), and education level. These factors were selected due to their established relevance in CRC research and their potential influence on patient behaviors and outcomes in tertiary prevention.

Age is a critical determinant of cancer risk, treatment tolerance, and survivorship experiences. Older adults often present with distinct comorbidities, immune and metabolic changes, and nutritional vulnerabilities that affect their ability to maintain healthy habits [7,9,19,20].

BMI is a modifiable factor closely associated with CRC prognosis: both obesity and undernutrition can impair treatment efficacy and quality of life by affecting inflammation, body composition, and metabolism [34,35,36,37].

Education level is a widely recognized social determinant of health, influencing health literacy, access to care, and behavioral patterns, including diet, physical activity, and substance use [38,39]. Together, these variables offer a relevant framework for stratifying CRC patients in order to explore differences in lifestyle behaviors that may inform more targeted preventive strategies.

Thus, this study aims to explore differences in dietary and lifestyle behaviors among CRC patients—stratified by age, BMI, and education level—in order to generate preliminary insights that may support more tailored strategies in tertiary prevention.

**Figure 1:**
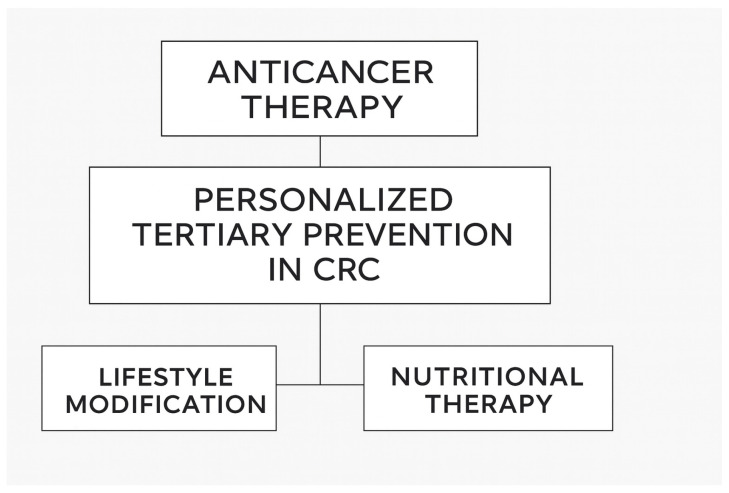
Model of personalized care for CRC patients.

A conceptual framework for individualized CRC care is presented in Figure 1.

## 2. Materials and Methods

### 2.1. Ethical Considerations

The study protocol was registered with the Bioethical Committee of the Medical University of Silesia in Katowice. The Committee stated that “the project does not meet the criteria of a medical experiment under the applicable law and does not require assessment by the bioethical committee” (decision no BNW/NWN/0052/KB/145/I/24). In accordance with this decision, written informed consent was not required under national regulations. However, verbal informed consent was obtained from all participants prior to data collection, in line with the principles of the Declaration of Helsinki (2013). Participation was entirely voluntary and based on informed decision-making. Before enrollment, all participants received full information about the study. To ensure confidentiality, pseudonymization was applied; identifying data were processed in a way that prevented participant identification without access to a separate reidentification key.

### 2.2. Study Group and Methodology

This study was designed as an exploratory, hypothesis-generating analysis aimed at identifying stratified patterns in dietary and lifestyle behaviors among CRC patients.

The study included 202 patients diagnosed with colorectal adenocarcinoma, comprising 119 women and 83 men, aged between 40 and 84 years. Diagnoses were based on the clinical staging system of the 9th edition of the Union for International Cancer Control/American Joint Committee on Cancer (UICC/AJCC). All patients were undergoing active anticancer treatment at the time of enrollment. Participants were recruited from the oncology departments of two multidisciplinary hospitals in the Silesia region of Poland, where the study was carried out. These hospitals are large, urban referral centers serving a socioeconomically and demographically diverse population, enhancing the generalizability of the study findings.

To enhance sample size, purposive recruitment was complemented by snowball sampling, enabling the inclusion of patients from community settings who met the inclusion criteria (shown below). Given the study’s specific focus on behavioral profiles in tertiary prevention, random sampling was not applied, as the primary aim was not population generalization but rather identification of relevant subgroup differences within the *CRC* population.

Participants were consecutively enrolled during a multi-month recruitment phase preceding data analysis.

*Inclusion criteria:*

- Histologically confirmed diagnosis of CRC, regardless of clinical stage;
- Age 18 years or older;
- Oral nutritional intake;
- Verbal consent to participate.

*Exclusion criteria:*

- Failure to meet inclusion requirements (e.g., diagnosis of non-CRC malignancies);
- Age under 18 years;
- Enteral or parenteral nutrition;
- Lack of consent;
- Incomplete or incorrect completion of the research tool;
- Withdrawal from the study.

### 2.3. Research Tool

The development process began with an initial pool of questions based on a comprehensive review of relevant literature. Data were collected using an original, anonymous questionnaire developed specifically for this study. The questionnaire was administered exclusively in a paper format during in-person medical appointments. This approach aimed to ensure accessibility, particularly for elderly participants and individuals with limited digital literacy, thereby enhancing response rates and data reliability across diverse demographic groups. The questionnaire was validated through two pilot tests involving 30 participants, conducted 30 days apart, and refined based on expert feedback and participant comprehension.

During the validation process, items related to dietary and lifestyle behaviors in *CRC* patients were critically reviewed. Two questions—comparing dietary and lifestyle patterns and nutritional problems before and during cancer—were removed due to participant confusion regarding their interpretation.

Responses were categorized as “high” or “low” based on median score distributions for each dimension. The final version of the questionnaire included tabular, open-ended, semi-open, and both single- and multiple-choice closed-ended questions. The first section addressed sociodemographic and clinical data, followed by the core section focusing on the study’s primary objectives.

### 2.4. Statistical Analysis

Data were analyzed using Statistica for Windows version 13.3. The chi-square (χ^2^) test was employed to examine relationships between categorical variables. Specifically, statistical associations were tested between dietary and lifestyle behavior indicators (including nutritional difficulties, dietary habits, physical activity levels, and sleep quality) and key patient characteristics: age group, body mass index (BMI), and educational level. Analyses were conducted both for the total study population and within each of the stratified subgroups.

Results are presented as frequencies and percentages. A *p*-value of <0.05 was considered statistically significant. Due to the broad scope of the analysis and the exploratory nature of the study, only statistically significant associations are reported in this manuscript to enhance clarity and focus. No formal correction for multiple comparisons was applied, as the primary goal was to identify potentially relevant patterns rather than test predefined hypotheses. Therefore, findings should be interpreted as hypothesis-generating and form a basis for future confirmatory research.

### 2.5. Research Design

**Figure 2:**
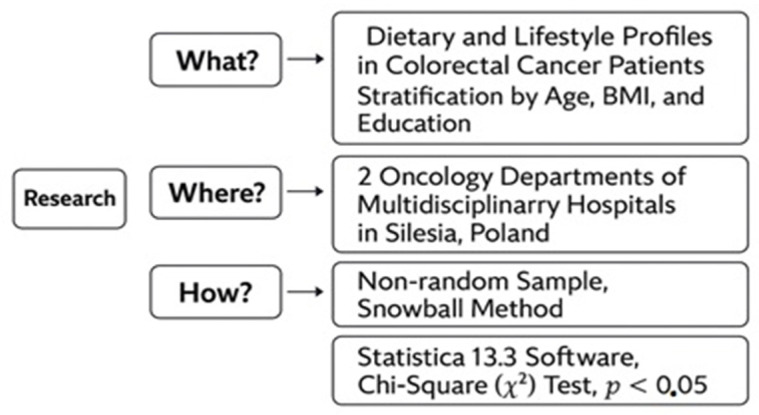
Schematic overview of the research design.

The structure of the research model is illustrated in Figure 2.

## 3. Results

### 3.1. Participant Characteristics

A total of 202 patients diagnosed with *CRC* included in the study. The study group consisted of 119 women (58.9%) and 83 men (41.1%). Most participants were classified as middle-aged (69.3%, *n* = 141), while the remaining 30.2% (*n* = 61) were elderly. Most of the study participants had secondary education (*n* = 96; 47.5%) and were overweight (*n* = 110; 54.4%) (Table 1).

Most patients were diagnosed 7–12 months prior (45.5%) or over a year earlier (23.3%). The predominant treatment was surgery combined with chemotherapy (60.4%), followed by chemotherapy alone (18.8%). Multimodal therapies, including radiotherapy or targeted agents, were less common (Table 2).

### 3.2. Dietary and Lifestyle Behaviors by Age

Significant differences in dietary and lifestyle behaviors were observed between middle-aged and elderly participants. Vegetable consumption several times per day was more frequent among elderly patients (52.5% vs. 40.4%), whereas legume consumption a few times per week was slightly more common among middle-aged patients (23.4% vs. 19.7%) (*p* = 0.03 and *p* = 0.04, respectively). Fast-food consumption was also more common in the middle-aged group (*p* = 0.03). Infrequent alcohol consumption (defined as “once a month or less”) was more common among elderly participants, while the proportion of never-smokers was slightly higher in the middle-aged group (33.3% vs. 32.8%) (*p* = 0.0009 and *p* = 0.04, respectively) (Table 3). Although differences were small, middle-aged participants showed slightly higher prevalence of never-smoking (33.3% vs. 32.8%) and reported more frequent consumption of fast food, while elderly participants more commonly reported infrequent alcohol use (once a month or less) (*p* = 0.0009 and *p* = 0.04, respectively).

### 3.3. Dietary Habits by Body Mass Index (BMI)

Dietary variables analyzed across BMI categories focused on meat consumption patterns and sugar intake, which were considered particularly relevant to adiposity-related dietary behaviors. This differs from the age-stratified analysis, which emphasized broader lifestyle and nutritional factors. Due to the very small number of participants in the underweight category (*n* = 2), this group was excluded from BMI-stratified comparisons to preserve statistical validity.

Frequency of meat consumption and thermal preparation methods varied significantly by BMI category. Overweight and obese participants were more likely to consume meat frequently and preferred frying and boiling/steaming (*p* = 0.02 and *p* = 0.03, respectively). Sugar and sweets consumption was also significantly associated with BMI (*p* = 0.03), with daily and weekly intake being more common in overweight and obese participants (Table 4).

### 3.4. Substance Use by Educational Level

Alcohol consumption and smoking status differed significantly by educational attainment. Infrequent alcohol consumption (defined as “once a month or less”) was most commonly reported among participants with secondary education (82.3%), followed by those with higher (75.4%) and vocational education (73.3%) (*p* = 0.004). A higher proportion of never-smokers was observed among patients with higher education (45.9%) compared to those with secondary (34.4%) and vocational education (13.3%) (*p* = 0.02) (Table 5). Current smoking was most prevalent among participants with vocational education.

## 4. Discussion

During oncological treatment, both dietary intake and physical activity are frequently compromised due to the physical and systemic burden associated with therapy. Common adverse effects—such as gastrointestinal symptoms, anemia, or treatment-induced fatigue—may limit patients’ ability to maintain adequate nutrition and remain physically active. These disruptions can undermine key components of tertiary prevention, particularly in terms of preserving muscle mass, functional capacity, and overall treatment tolerance [30].

In this context, understanding how individual characteristics relate to lifestyle behaviors becomes clinically relevant. Our study aimed to explore whether such behaviors differ by age, body mass index (BMI), and educational level among patients with colorectal cancer (CRC). While each of these variables has been independently associated with health-related behaviors in previous epidemiological studies [7,9,10,15], the descriptive nature of our analyses does not allow for assessment of their combined or interactive effects. Nevertheless, the behavioral patterns observed here may provide a basis for future hypothesis-driven research that further examines how these factors operate within the framework of tertiary prevention.

Given the cross-sectional design, limited subgroup sizes, and lack of adjustment for multiple comparisons, our findings should be interpreted with caution. They are exploratory in nature and intended primarily to generate hypotheses. Still, the emerging trends offer preliminary insight into how sociodemographic and clinical characteristics may shape behavioral responses during cancer care.

### 4.1. Age-Dependent Behavioral Patterns

Middle-aged patients in our study demonstrated a mixed behavioral profile, characterized by both increased consumption of vegetables and legumes as well as a higher intake of fast food. While this duality is difficult to interpret definitively, it may reflect competing influences such as occupational stress, inconsistent symptom awareness, or time constraints related to food preparation. Recent studies suggest that only a minority of middle-aged cancer survivors adhere consistently to multiple recommended lifestyle behaviors, with dietary habits and physical activity levels being among the least frequently met guidelines, despite widespread access to health information and resources [40,41].

Older adults more frequently reported abstinence from alcohol and tobacco, which may be shaped by generational norms or reflect longer-term adaptation to chronic disease. At the same time, their lower vegetable intake may be linked to age-associated barriers—such as reduced appetite, oral health issues, physical limitations, and cognitive decline—factors well documented in geriatric oncology literature [42,43,44,45]. These multifactorial challenges can substantially undermine diet quality and must be carefully considered when tailoring nutritional interventions for older cancer patients. Importantly, given the cross-sectional nature of this analysis, observed associations should be interpreted with caution and regarded as a basis for future hypothesis-driven research.

### 4.2. BMI and Dietary Habits

Overweight and obese CRC patients more frequently consumed meat, sweets, and fried foods—dietary patterns associated with systemic inflammation and poorer clinical outcomes [46,47]. According to many authors, excess body fat is a well-established carcinogenic factor, particularly in colorectal cancer development [34,47]. However, beyond its etiological role, obesity may also adversely affect clinical outcomes by promoting low-grade systemic inflammation, insulin resistance, and metabolic dysregulation [35,36,48]. Importantly, high BMI does not exclude the presence of malnutrition. A growing body of evidence highlights the phenomenon of sarcopenic obesity and hidden malnutrition—conditions characterized by increased adiposity with concurrent muscle mass loss, micronutrient deficiencies, or impaired functional status, which may lead to reduced chemotherapy tolerance, increased toxicity, and poorer prognosis [37,49,50].

The frequent use of frying as a cooking method observed in this group reflects broader global dietary shifts toward ultra-processed and pro-inflammatory foods. These patterns have been associated with insulin resistance, gut microbiota alterations, and a potential role in colorectal cancer progression [51,52]. The frequent use of frying as a cooking method observed in this group may reflect preferences for energy-dense and potentially pro-inflammatory foods. However, since the study did not assess the consumption of ultra-processed foods or inflammatory markers, such interpretations remain speculative and should be viewed as preliminary. Therefore, BMI should not only be considered a risk marker but also a clinically relevant factor requiring comprehensive assessment. Incorporating body composition analysis and routine nutritional screening may improve risk stratification and support personalized dietary counseling and therapeutic planning [53].

### 4.3. Education and Health Literacy

Higher educational attainment was associated with lower reported use of alcohol and tobacco, which is consistent with previous evidence linking education to better health literacy and greater engagement in self-care behaviors [38,39]. Although our study did not assess adherence to specific clinical guidelines, the observed associations suggest that educational background may influence behaviors such as diet and physical activity, both of which are considered important components of tertiary prevention.

Patients with lower levels of education may benefit from tailored communication strategies aimed at improving comprehension and encouraging behavior change. The literature supports the use of simplified written instructions, visual tools (e.g., infographics and pictograms), culturally adapted materials, and multimedia formats to enhance understanding and engagement. Such approaches have been highlighted by Campbell et al. and Schmitz et al., who emphasize the role of individualized survivorship care and clear health communication in supporting long-term behavioral outcomes among cancer survivors [54,55,56].

### 4.4. Clinical Implications and Recommendations

Recent interventional studies in colorectal cancer (CRC) patients suggest that personalized nutritional and lifestyle programs may improve adherence and patient-reported outcomes. For example, a randomized controlled trial in CRC survivors demonstrated that individualized nutrition counseling with follow-up was associated with improved nutritional status and quality of life compared to standard care [57]. Additionally, structured interventions tailored to patient needs and delivered via multimedia platforms have shown variable but promising results in enhancing compliance and preserving functional capacity in oncological settings [58,59]. Current clinical guidelines emphasize the need to tailor supportive interventions according to age, BMI, and comorbidities, particularly during active anticancer treatment [60].

The present study included patients undergoing active anticancer therapy, with varying durations since diagnosis and treatment modalities. These clinical factors likely influenced dietary and lifestyle behaviors due to symptom burden, treatment-related side effects, and psychological stress. Therefore, it is plausible that the behaviors observed in this group were shaped, at least in part, by disease stage and treatment type (e.g., chemotherapy vs. surgery), which should be taken into account when interpreting behavioral patterns and designing future interventions [61,62].

Although our findings are exploratory and intended to generate hypotheses, they point toward the potential utility of a multidimensional approach to tertiary prevention in CRC. Specifically, future strategies may benefit from individualization based on sociodemographic, physical, and psychosocial parameters. While standardized protocols offer scalability, they may fail to address the complex and nuanced needs of older, overweight, or socially disadvantaged patients.

Based on observed trends, the following patient-specific considerations could inform the design of future personalized supportive care programs:

- Middle-aged patients: Emphasis on behavioral counseling to reduce fast-food and stimulant consumption, alongside guidance for balanced dietary planning.
- Elderly patients: Interventions addressing functional limitations—such as meal assistance, oral health care, and appetite stimulation—to mitigate the risk of malnutrition.
- Overweight and obese patients: Nutritional support aimed at reducing energy-dense food intake while preventing micronutrient deficiencies; consideration of high-fat cooking methods (e.g., frying) within broader dietary patterns.
- Patients with lower educational attainment: Multimodal educational strategies tailored to health literacy levels, aimed at fostering long-term behavioral change.

We suggest that future studies explore the integration of interdisciplinary care teams—including dietitians, psycho-oncologists, physiotherapists, and social workers—within routine oncological follow-up. Such models may enhance adherence, reduce treatment-related toxicity, and improve patients’ quality of life.

Ultimately, scaling up stratified supportive care strategies within national cancer control programs may help align healthcare resources with patient-specific profiles. However, confirmatory studies are needed to validate these exploratory findings and inform evidence-based clinical guidelines.

### 4.5. Future Research Directions

Future research should aim to generate and consolidate knowledge across the interconnected domains of oncological nutrition, psycho-oncology, quality of life, and tertiary prevention in colorectal cancer, as well as gastrointestinal cancers more broadly. There is a growing need for comprehensive, multidisciplinary studies that explore the complex interplay between patients’ individual characteristics, clinical parameters, and health-related behaviors.

In particular, future interventional studies should investigate how personalized nutritional and lifestyle interventions interact with laboratory results, anthropometric measurements, and psychosocial determinants of health. Understanding these relationships may help uncover new correlations and causal mechanisms that could inform more precise and effective tertiary prevention strategies.

A critical area requiring further exploration is the use of nutritional assessment as a screening tool in this patient population. This approach is supported by current guidelines, which emphasize early identification of malnutrition risk. Systematically comparing nutritional status with other clinical, functional, and psychosocial parameters may enable early identification of at-risk individuals and support timely, individualized interventions. These elements should form the basis of an integrated model of care that addresses both physical and psychological dimensions of patient well-being.

Despite the recognized importance of individualized oncology care, current research still falls short in addressing the multifactorial nature of patient needs, particularly in colorectal cancer populations. Expanding research in these domains is essential to bridge existing knowledge gaps and translate findings into actionable clinical practice. Future investigations should focus on identifying predictors of treatment response, quality of life trajectories, and behavioral adherence, all within a personalized framework that reflects the heterogeneity of cancer patients.

## 5. Strengths and Limitations of the Study

### 5.1. Strengths

This study has several important strengths. Most notably, it addresses tertiary prevention in CRC—a domain that remains underrepresented in clinical research despite its essential role in survivorship care and long-term quality of life.

The study demonstrates a novel and unique approach by examining dietary and lifestyle behaviors in relation to key demographic variables such as age, BMI, and educational level. Few existing studies have explored these correlations simultaneously, highlighting a significant gap in the literature that this study begins to address.

The use of a relatively large and demographically diverse sample (*n* = 202) allowed for robust subgroup analyses, providing deeper insight into behavioral differences among CRC patients.

Nevertheless, the analysis was descriptive and exploratory in nature, without the use of multivariate statistical models.

Furthermore, the use of a validated questionnaire, refined through pilot testing and expert consultation, ensured methodological rigor and reliable self-reported data collection.

### 5.2. Limitations

This study was exploratory and hypothesis-generating in nature; thus, its findings should be interpreted with caution and not generalized without confirmation from further research.

Although the sample included participants with varied age and BMI profiles, it was geographically restricted to a single region in Poland, which may limit its generalizability to other populations with different cultural or healthcare systems.

Socioeconomic status was approximated by educational level, as data on income or occupation were not collected, which may not fully capture economic influences on health behaviors. Employment status and work-related stress were also not assessed but are planned for inclusion in future studies.

The snowball sampling method may have introduced selection bias, potentially overrepresenting specific groups.

The questionnaire did not include detailed mental health assessment, which is a relevant factor in shaping lifestyle behaviors.

The cross-sectional design precludes causal inferences, and the use of self-reported data may be affected by recall or social desirability bias.

Finally, clinical variables were limited to time since diagnosis and treatment type; future studies will include more comprehensive clinical data to enhance the depth of analysis.

## 6. Conclusions

This exploratory study suggests that dietary and lifestyle behaviors among colorectal cancer (CRC) patients may differ based on age, BMI, and educational level. The observed patterns indicate the potential value of individualized, demographically sensitive approaches to tertiary prevention. While the findings highlight the relevance of nutritional screening and tailored support within oncological care, they should be interpreted with caution due to the study’s limitations. Further research is warranted to confirm these associations and to support the development of evidence-based interventions aimed at improving long-term outcomes and quality of life in diverse CRC populations.

## Figures and Tables

**Table 1:** Sociodemographic characteristics of the study population.

		*n* = 202 (*n*%)
**Sex**	*Female*	119 (58.9%)
	*Male*	83 (41.1%)
**Age**	*Middle-aged (40–64 years old)*	141 (69.3%)
	*Elderly (≥65 years old)*	61 (30.2%)
**Education Level**	*Basic*	0 (0.0%)
	*Vocational*	45 (22.3%)
	*Secondary*	96 (47.5%)
	*Higher*	61 (30.2%)
**Body Mass Index (BMI)**	*Underweight (<18.5 kg/m^2^)*	2 (1.0%)
	*Normal (18.5–24.9 kg/m^2^)*	62 (30.7%)
	*Overweight (25.0–29.9 kg/m^2^)*	110 (54.4%)
	*Obesity (≥30 kg/m^2^)*	28 (13.9%)

**Table 2:** Clinical characteristics of the study population.

		*n* = 202 (*n*%)
**Time since diagnosis**	*1–3 months*	12 (5.9%)
	*4–6 months*	51 (25.3%)
	*7–12 months*	92 (45.5%)
	*>12 months*	47 (23.3%)
**Type of treatment received**	*Surgery only*	17 (8.4%)
	*Surgery + chemotherapy + targeted therapy*	5 (2.5%)
	*Chemotherapy + targeted therapy*	9 (4.5%)
	*Chemotherapy only*	38 (18.8%)
	*Surgery + chemotherapy*	122 (60.4%)
	*Surgery + chemotherapy + radiotherapy*	11 (5.4%)

**Table 3:** Frequency of selected dietary and lifestyle behaviors by age.

Frequency of Vegetables Consumption	Middle-Aged *n* = 141 (*n*%)	Elderly *n* = 61 (*n*%)	*p*-Value
*A few times a day*	57 (40.4%)	32 (52.5%)	0.03
*Once a day*	62 (44.0%)	24 (39.3%)	
*A few times a week*	20 (14.2%)	2 (3.3%)	
*Once a week*	1 (0.7%)	2 (3.3%)	
*A few times a month*	1 (0.7%)	0 (0.0%)	
*Once a month or less*	0 (0.0%)	1 (1.6%)	
**Frequency of Legumes** **Consumption**			
*A few times a day*	0 (0.0%)	0 (0.0%)	0.04
*Once a day*	0 (0.0%)	0 (0.0%)	
*A few times a week*	4 (2.8%)	0 (0.0%)	
*Once a week*	5 (3.6%)	2 (3.3%)	
*A few times a month*	33 (23.4%)	12 (19.7%)	
*Once a month or less*	99 (70.2%)	47 (77.0%)	
**Frequency of Fast-Food** **Consumption**			
*Every day*	0 (0.0%)	0 (0.0%)	0.03
*A few times a week*	0 (0.0%)	0 (0.0%)	
*Once a week*	4 (2.8%)	0 (0.0%)	
*A few times a month*	10 (7.1%)	1 (1.6%)	
*Once a month or less*	127 (90.1%)	60 (98.4%)	
**Frequency of Alcohol Consumption**	0 (0.0%)	0 (0.0%)	
*Every day*	0 (0.0%)	0 (0.0%)	0.0009
*A few times a week*	8 (5.7%)	4 (6.6%)	
*Once a week*	10 (7.1%)	1 (1.6%)	
*A few times a month*	19 (13.5%)	2 (3.3%)	
*Once a month or less*	104 (73.8%)	54 (88.5%)	
**Smoking Status**	0 (0.0%)	0 (0.0%)	
*Currently smoking*	14 (9.9%)	4 (6.6%)	0.04
*Not currently smoking,* *but smoked before the diagnosis*	71 (50.4%)	37 (60.7%)	
*Not currently smoking,* *but smoked during the disease*	9 (6.4%)	0 (0.0%)	
*Never smoked*	47 (33.3%)	20 (32.8%)	

**Table 4:** Dietary habits by body mass index (BMI).

Frequency of Meat Consumption	Normal Weight *n* = 62 (*n*%)	Overweight *n* = 110 (*n*%)	Obesity *n* = 28 (*n*%)	*p*-Value
*Every day*	4 (6.5%)	7 (6.4%)	7 (25.0%)	0.02
*A few times a week*	47 (75.8%)	92 (83.6%)	17 (60.7%)	
*Once a week*	9 (14.5%)	10 (9.1%)	2 (7.1%)	
*A few times a month*	0 (0.0%)	0 (0.0%)	1 (3.6%)	
*Once a month or less*	2 (3.2%)	1 (0.9%)	1 (3.6%)	
**Thermal Methods** **of Meat Preparation**				
*None (raw meat)*	0 (0.0%)	0 (0.0%)	0 (0.0%)	0.03
*Boiling/Steaming*	25 (41.0%)	52 (47.7%)	12 (44.4%)	
*Frying*	11 (18.0%)	24 (22.0%)	11 (40.7%)	
*Roasting*	5 (8.2%)	2 (1.8%)	3 (11.1%)	
*Grilling*	0 (0.0%)	0 (0.0%)	0 (0.0%)	
*Stewing*	20 (32.8%)	31 (28.4%)	1 (3.7%)	
**Frequency of Sugar** **and Sweets Consumption**				
*Every day*	11 (17.7%)	25 (22.7%)	12 (42.9%)	0.03
*A few times a week*	22 (35.5%)	49 (44.6%)	8 (28.6%)	
*Once a week*	6 (9.7%)	9 (8.2%)	0 (0.0%)	
*A few times a month*	11 (17.7%)	20 (18.2%)	5 (17.9%)	
*Once a month or less*	12 (19.4%)	7 (6.4%)	3 (10.7%)	

**Table 5:** Alcohol consumption and smoking status by education level.

Frequency of Alcohol Consumption	Vocational *n* = 45 (*n*%)	Secondary *n* = 96 (*n*%)	Higher *n* = 61 (*n*%)	*p*- Value
*Every day*	0 (0.0%)	0 (0.0%)	0 (0.0%)	0.004
*A few times a week*	4 (8.9%)	8 (8.3%)	0 (0.0%)	
*Once a week*	4 (8.9%)	3 (3.1%)	4 (6.6%)	
*A few times a month*	4 (8.9%)	6 (6.3%)	11 (18.0%)	
*Once a month or less*	33 (73.3%)	79 (82.3%)	46 (75.4%)	
**Smoking Status**				
*Currently smoking*	7 (15.6%)	8 (8.3%)	3 (4.9%)	0.02
*Not currently smoking, but smoked before the diagnosis*	29 (64.4%)	52 (54.2%)	27 (44.3%)	
*Not currently smoking,* *but smoked during the disease*	3 (6.7%)	3 (3.1%)	3 (4.9%)	
*Never smoked*	6 (13.3%)	33 (34.4%)	28 (45.9%)	

## Data Availability

Data is contained within the article.
